# Supplementary material for: Splice-Junction-Based Mapping of Alternative Isoforms in the Human Proteome
Source: Cell Rep. Author manuscript; Available in PMC 2020 Jan 15. (PMC6961840; doi:10.1016/j.celrep.2019.11.026)
Supplement: 2 [file NIHMS1546469-supplement-2.zip › DF1/PXD006675/PulmonaryVein/PulmonaryVein_20_BBS1_EGQSAPLLSAHVNMPGSEGLAAPNRPLNPE.pdf]

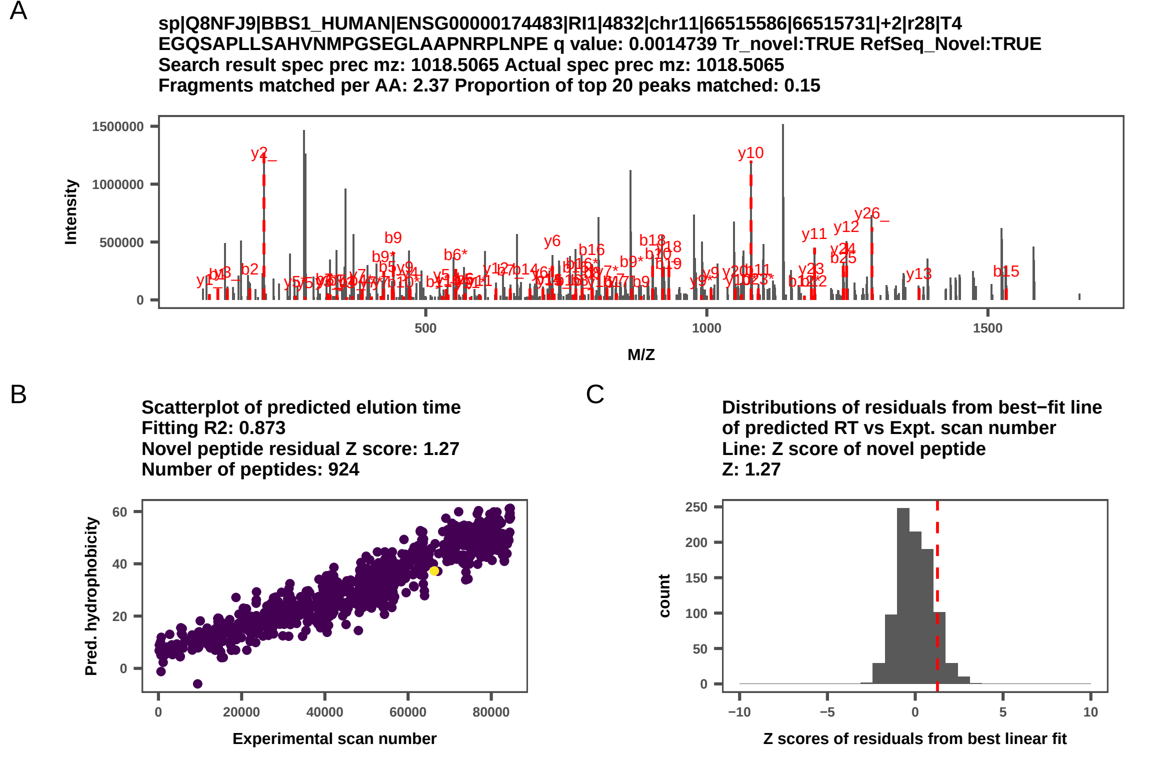
A
sp|Q8NFJ9|BBS1_HUMAN|ENSG00000174483|RI1|4832|chr11|66515586|66515731|+2|r28|T4
EGQSAPLLSAHVNMPGSEGLAAPNRPLNPE q value: 0.0014739 Tr_novel:TRUE RefSeq_Novel:TRUE
Search result spec prec mz: 1018.5065 Actual spec prec mz: 1018.5065
Fragments matched per AA: 2.37 Proportion of top 20 peaks matched: 0.15
Intensity
1500000
1000000
500000
0
500
1000
1500
M/Z
y2_
y10
y26_
b9
b9*
b6*
y6
b16
b18
y11
y12
y24
b25
y23
y13
b15
b2
B
Scatterplot of predicted elution time
Fitting R2: 0.873
Novel peptide residual Z score: 1.27
Number of peptides: 924
Pred. hydrophobicity
60
40
20
0
0
20000
40000
60000
80000
Experimental scan number
C
Distributions of residuals from best−fit line
of predicted RT vs Expt. scan number
Line: Z score of novel peptide
Z: 1.27
count
250
200
150
100
50
0
−10
−5
0
5
10
Z scores of residuals from best linear fit
